# Safety and efficacy of protease inhibitor based combination therapy in a single-center “real-life” cohort of 110 patients with chronic hepatitis C genotype 1 infection

**DOI:** 10.1186/1471-230X-14-87

**Published:** 2014-05-05

**Authors:** Malte H Wehmeyer, Friederike Eißing, Sabine Jordan, Claudia Röder, Annette Hennigs, Olaf Degen, Anja Hüfner, Sandra Hertling, Stefan Schmiedel, Martina Sterneck, Jan van Lunzen, Ansgar W Lohse, Julian Schulze zur Wiesch, Stefan Lüth

**Affiliations:** 1 I. Medizinische Klinik und Poliklinik, Universitätsklinikum Hamburg-Eppendorf, Martinistr. 52, Hamburg 20246, Germany; 2 Ambulanzzentrum des UKE, Fachbereich Infektiologie, Hamburg Germany

**Keywords:** Boceprevir, Serious adverse events, SAE, Side effects, Sustained virological response, SVR, Telaprevir

## Abstract

**Background:**

The combination of boceprevir or telaprevir with peginterferon-alfa and ribavirin for the treatment of patients infected with HCV genotype 1 has led to significantly increased rates of sustained virological response (SVR) in phase III trials. There is only limited data regarding the safety and efficacy in a “real-life” cohort.

**Methods:**

We analyzed a cohort of 110 unselected HCV patients who started triple therapy from September 2011 to February 2013 by chart review with focus on the individual course of treatment, complications and outcome. We excluded 8 patients from analysis because of HIV-coinfection (N = 6) or status post liver transplant (N = 2). Importantly, 41 patients displayed F3 or F4 fibrosis, 10 patients had a history of treatment with protease/polymerase inhibitors and 15 patients were prior partial- or null-responder.

**Results:**

SVR12 was achieved in 62 of the 102 patients (60.8%). A high rate of serious adverse events (N = 30) was observed in 22 patients including 2 fatalities in cirrhotic diabetes patients. Age >50 years, liver cirrhosis, bilirubin >1.1 mg/dl (P < 0.01, each), platelets <100,000/μl (P = 0.01), ASAT >100 U/l (P = 0.03) and albumin ≤35 g/l (P = 0.04) at baseline were associated with occurence of a SAE.

**Conclusions:**

The frequency of SVR in a “real-life” treatment setting is slightly lower as compared to the results of the phase III trials for telaprevir or boceprevir. Importantly, we observed a high frequency of SAE in triple therapy, especially in patients with liver cirrhosis.

## Background

An estimated 170 million people are chronically infected with the hepatitis C virus (HCV) [1] and have an elevated risk for liver-related mortality [2]. Recently, introduction of the serine protease inhibitors (PI) boceprevir (BOC) and telaprevir (TPR) which are used in combination with peginterferon-alfa 2a or 2b (pegIFN) and ribavirin (RBV) has increased cure rates of patients with chronic HCV genotype 1 infection in the US, Canada and many European countries in phase III trials with sustained virological response (SVR) rates of 67 to 75% in treatment naive patients [3-5]. Even higher SVR rates have been achieved in patients with history of relapse following a previous therapy [6,7]. Interestingly, first “real-life” efficacy data revealed a significantly lower frequency of SVR [8]. On the other hand, treatment with a PI is associated with high rates of side-effects, such as fatigue, anemia and high-grade neutropenia [3-7]. Skin reactions and gastrointestinal disorders were frequently observed side-effects of TPR in the phase II and III trials [4,5,7].

However, the overall safety-profile of the PIs was acceptable in phase III trials [3-7], which included highly selected patients. Most recently, preliminary “real-life” data covering the first 12 to 16 weeks of therapy revealed considerably increased risk for severe and in some instances even lethal complications of PI-based treatment in cirrhotic patients [9,10]. The most common cause of death was sepsis, with staphylococcus being the most frequent causative organism [10].

Despite the approval of alternative direct acting antivirals (DAA) in Northern America and Europe, TPR and BOC have just arrived in many parts of the world. Given the increased likelihood of serious adverse events (SAE) provided by the preliminary reports on “real-life” data [9,10], we examined the outcomes and complications of triple therapy throughout the treatment course within our local “real-life”, difficult-to-treat cohort, which includes a number of patients with comorbidities, cirrhosis or previous DAA experience.

## Methods

### Study population and chart review

We analyzed clinical and laboratory data of 110 unselected patients who were chronically infected with HCV genotype 1 and in whom treatment with pegIFN, RBV and TPR or BOC was initiated from September 2011 to February 2013 at the viral hepatitis clinics of the University Medical Center Hamburg-Eppendorf, which is representative of a tertiary care referral center for antiviral HCV therapy in Germany. Liver transplant recipients (N = 2) and patients coinfected with human immunodeficiency virus were excluded (N = 6).

All patients received an abdominal ultrasound prior to the start of therapy. The grade of liver fibrosis was measured in the majority of patients by transient elastography (Fibroscan, Echosens, France) [11] or liver biopsy before the initiation of treatment (Table 1).

**Table 1:** Baseline characteristics

	**All patients**	**Telaprevir**	**Boceprevir**
	**(N = 102)**	**(N = 65)**	**(N = 37)**
	**N (%); median (range)**	**N (%); median (range)**	**N (%); median (range)**
**Male sex**	63 (62%)	43 (66%)	20 (54%)
**Exclusion criteria for appropriate phase III trials**	65 (64%)	42 (65%)	23 (62%)
**Treatment naïve**	47 (46%)	23 (35%)	24 (65%)
**Treatment experienced**	55 (54%)	42 (65%)	13 (35%)
*Relapse*	25 (25%)	18 (28%)	7 (19%)
*Null/partial response*	15 (15%)	13 (20%)	2 (5%)
*Breakthrough*	6 (6%)	5 (8%)	1 (3%)
*Discontinuation*^*§*^	5 (5%)	3 (5%)	2 (5%)
*Unknown outcome*	4 (4%)	3 (5%)	1 (3%)
**DAA experienced**	10 (10%)	9 (14%)	1 (3%)
**RVR**	38 (37%)	21 (32%)	17 (46%)
**Genotype**			
*Genotype 1a*	39 (38%)	20 (31%)	19 (51%)
*Genotype 1b*	53 (52%)	37 (57%)	16 (43%)
*No subtype provided*	9 (9%)	7 (11%)	2 (5%)
*Unknown*	1 (1%)	1 (2%)	0
**IL28B** (N = 70)			
*C/C*	16 (24%)	10 (20%)	6 (29%)
*C/T*	42 (60%)	32 (65%)	10 (48%)
*T/T*	12 (17%)	7 (14%)	5 (24%)
**Stage of fibrosis** (N = 92)			
*No or mild fibrosis (F0-F2)*	51 (55%)	32 (49%)	19 (59%)
*Bridging fibrosis (F3)*	12 (13%)	6 (10%)	6 (19%)
*Liver cirrhosis*	29 (32%)	22 (37%)	7 (22%)
*Hemoglobin [g/dl]*	14.7 (10.3-18.8)	14.7 (11.1-18.8)	14.6 (10.3-17.6)
*Leukocytes [x10^9/l]*	6.1 (2.7-13.1)	5.9 (2.7-13.1)	6.3 (3.9-12.4)
*Platelets [x10^9/l]*	188 (48–377)	180 (48–377)	203 (67–338)
*ASAT [U/l]*	50.5 (16–328)	52 (16–328)	44 (19–156)
*ALAT [U/l]*	75.5 (16–271)	87 (16–271)	72 (19–227)
*γGT [U/l]*	66.5 (25–1274)	67 (25–1274)	62 (25–217)
*Bilirubin [mg/dl]*	0.5 (0.2-2.2)	0.6 (0.3-2.2)	0.5 (0.2-1.4)
*Albumin [g/l]*	40 (25–50)	40 (25–47)	39 (30–50)
*Prothrombin time [INR]*	1.00 (0.90-3.29)	1.02 (0.90-3.29)	1.00 (0.92-2.33)
*Creatinine [mg/dl]*	0.8 (0.5-5.8)	0.8 (0.5-1.1)	0.8 (0.5-5.8)
**Viral load undetectable at**			
*EOT*	82 (80%)	51 (78%)	31 (84%)
*SVR12*	62 (61%)	40 (62%)	22 (59%)

[N = number; DAA = direct acting antivirals; RVR = rapid virological response; IL28B = interleukin-28B polymorphism; EOT = end of treatment; SVR12 = sustained virological response 12 weeks after last ribavirin dose; ^§^ = cessation of prior therapy due to side-effects].

Patient charts were analyzed regarding demographics, clinical data, HCV genotype, interleukin 28B (IL28B) rs12979860 polymorphism, as well as laboratory values and HCV viral load at different time points. The lower detection limit of the HCV PCR was 15 IU/ml (COBAS TaqMan HCV Qualitative, v2.0, Roche). The Child-Pugh score and the MELD score were assessed in all cirrhotic patients at baseline using the established formula [12,13].

### Statistical analysis

Variables of patients with SVR12 were compared with those of patients experiencing a treatment failure by univariate analysis using Fisher’s exact text, t-test (for variables with assumed Gaussian’ distribution, e.g. age) and Mann–Whitney-U-Test (for variables without assumed Gaussian’ distribution, e.g. laboratory values), respectively. The same analysis was conducted for patients suffering from predefined SAEs. Thresholds for continuous variables were defined according to the results from the CUPIC cohort [10] or by clinical judgement. Variables which reached P < 0.1 in univariate analysis were entered in a backward step logistic regression model.

The respective grading of laboratory events, adverse event definitions and virological definitions are described in the supplementary materials (text document, Additional file 1). All analyses were performed using SPSS Version 20. The figures were created using GraphPad Prism 4. The study was approved at the local ethics board (Ethik-Kommission der Ärztekammer Hamburg).

## Results

### Characterization of the study population

We describe here the detailed clinical course and treatment outcome of 102 patients who started triple therapy from September 2011 to February 2013 at our university viral hepatitis clinics. Baseline characteristics of all patients are summarized in Table 1. Fibroscan or liver biopsy was performed in 92 patients (90.2%) and diagnosis of bridging fibrosis (F3) or cirrhosis (F4) was established in 41 patients (40.2%). The 10 remaining patients, who did not receive a transient elastography or a liver biopsy prior to the HCV therapy, did not have any laboratory or sonographical evidence for high grade fibrosis or cirrhosis, respectively. IL28B polymorphism was assessed in 70 patients (68.6%), of whom 16 individuals displayed the favorable C/C IL28 haplotype (22.9%). Fifty five patients (53.9%) were HCV treatment experienced (25 patients with a prior relapse, 15 patients with prior partial- or null-response), including 10 patients who previously received a DAA based therapy in clinical trials. The exclusion criteria for registration trials for TPR or BOC [3-7] were met by 65 patients (64%, e.g. history of hepatocellular carcinoma, history of stem cell transplantation, renal dialysis, Crohn’s disease, thalassaemia major, autoimmune hepatitis and primary biliary cirrhosis). Twenty patients (19.6%) suffered from concomittant psychiatric disorders such as major depression (N = 18), anxiety disorder (N = 2), borderline psychosis (N = 1), post traumatic stress disorder (N = 1) and anorexia (N = 1) and were treated with psychotropics. Additionally, 10 patients were DAA experienced as participants of several phase II and III trials performed at our center.

**Figure 1:**
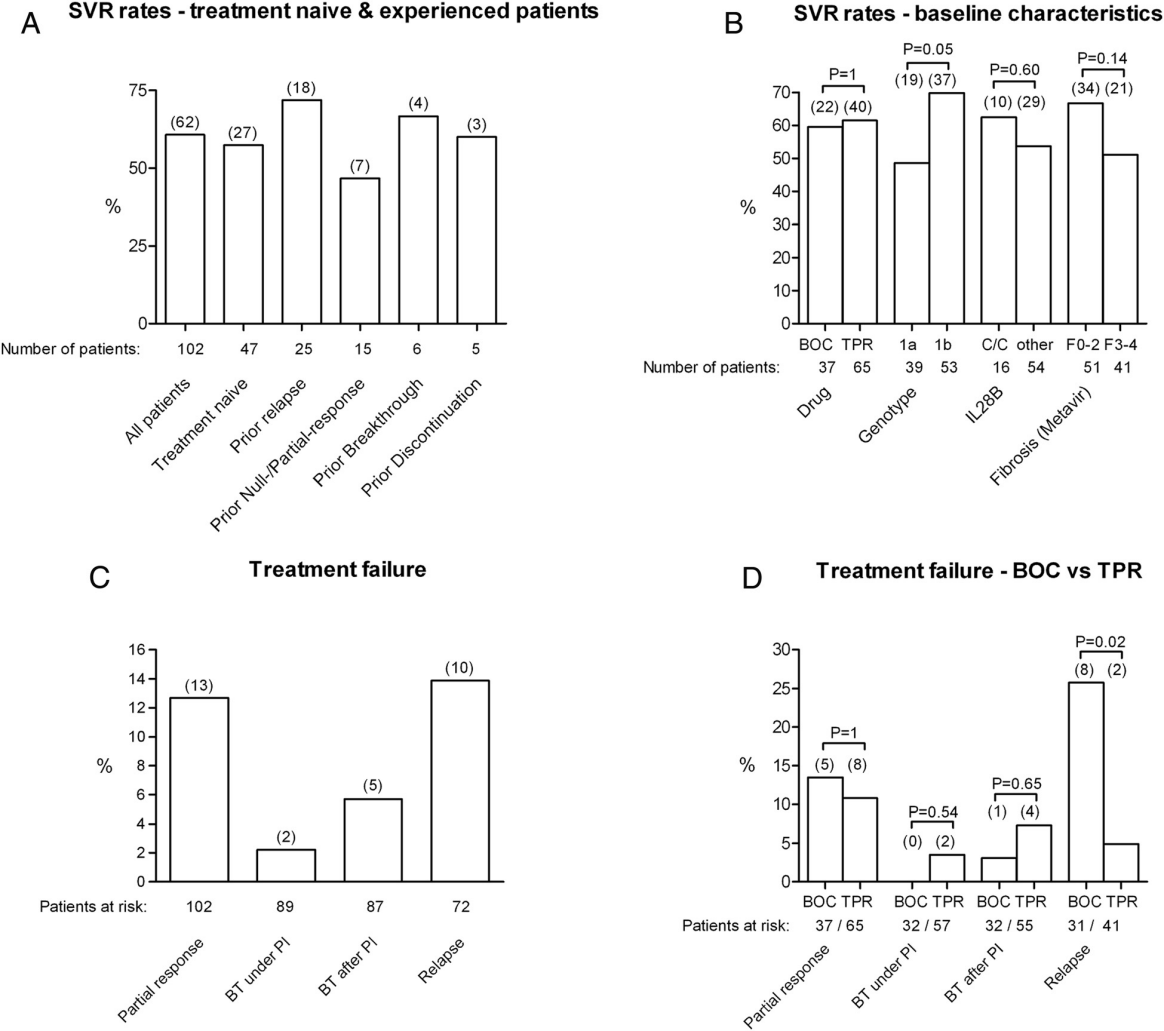
**Efficacy of triple therapy.** SVR rates for different subgroups are displayed in **A** and **B**, a characterization of treatment failures for the total study population **[C]** and for BOC versus TPR **[D]** are given, too. Patients who died after discontinuation of therapy (N = 3) and patients who were lost to follow up (N = 8) were not regarded as being at risk for relapse in **C** and **D**. [SVR = sustained virological response; BOC = boceprevir; TPR = telaprevir; IL28B = interleukin 28B polymorphism; BT = breakthrough; PI = protease inhibitor].

**Figure 2:**
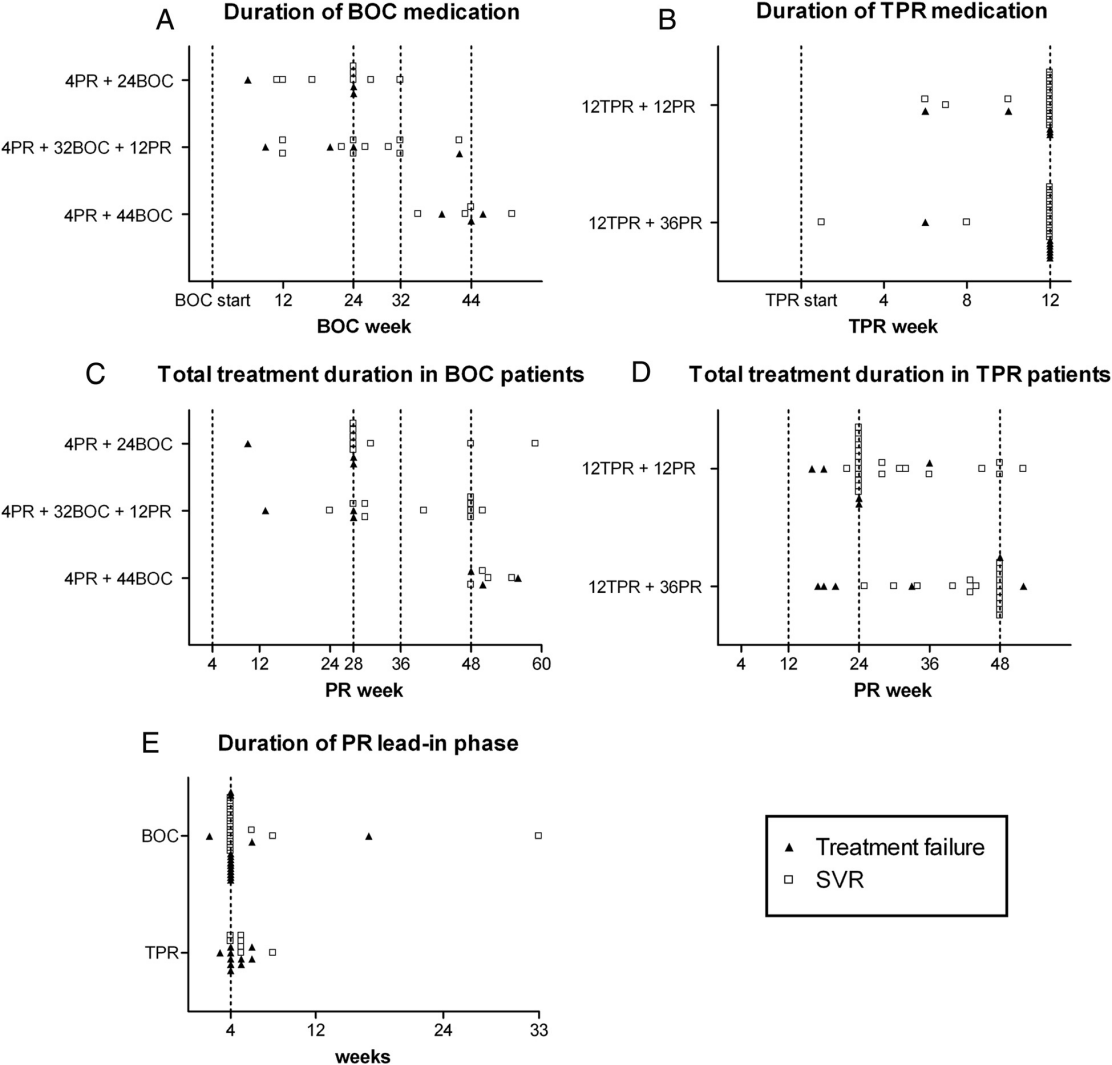
**Individualized courses of treatment in our “real-life” cohort.** Patients are grouped according to the respective guidelines [14,15]. Each symbol represents one patient. Patients with partial response or breakthrough under BOC/TPR are not depicted in **A** and **B**, patients with partial response or breakthrough at any time are not depicted in **C** and **D**. All BOC patients and TPR patients who received a dual lead-in phase prior to BOC/TPR are included in **E**. [BOC = boceprevir; TPR = telaprevir; PR = pegylated interferon and ribavirin; SVR = sustained virological response; EOT = patient concluded therapy, but is short of 12 weeks after last ribavirin dose].

In 15 patients virologic failure occured during PI treatment (Figure 1). The PI was discontinued early in 25 of the remaining 87 patients (28.7%) for various reasons (e.g. patient’s wish, side-effects, provider’s individual decision; Figure 2). After PI withdrawal, 5 additional patients experienced a viral breakthrough on dual therapy (Figure 1) and 24 of the remaining 82 patients (29.3%) discontinued pegIFN and RBV prematurely as compared to the guidelines for PI based therapy [14,15] (Figure 2). Seventeen patients treated with TPR (26.2%) and all patients treated with BOC received a lead-in phase with pegIFN and RBV prior to triple therapy (mean duration 4.7 weeks (TPR, standard deviation (SD) = 1.2) and 5.3 weeks (BOC, SD = 5.2), Figure 2). The rationale for starting therapy with a lead-in phase in TPR patients was to avoid the administration of a PI after a possible RVR under pegIFN/RBV [14].

As of now (April 2014), 7 patients (6.9%) are lost to follow-up and are therefore regarded as treatment failures in this analysis.

### Efficacy of triple therapy

Overall, 62 patients (60.8%) were successfully treated and achieved a SVR12. As expected, prior relapsers displayed the highest SVR rate of 72% and prior partial- or null-responders were less likely to achieve SVR12 (46.7%). SVR12 was achieved by 57.4% of treatment naive patients (including cirrhotics) and by 51.2% of patients with bridging fibrosis or liver cirrhosis (Figure 1). Finally, SVR 12 was achieved by 5/10 DAA experienced patients (see figure in Additional file 2, which includes an overview of the treatment regimen and clinical or virological outcome of the trial and the course of therapy with TPR or BOC for each DAA experienced patient, as well as the course of therapy of patients with special comorbidities. Further information on the clinical history of the DAA experienced patients are shown in the Additional file 1).

The HCV subtype was not significantly associated with SVR12 in our study. However, patients infected with HCV genotype 1a displayed an odds ratio (OR) of 0.4 to achieve SVR12 (95% confidence interval (95%CI) 0.2-1.0, *P* = 0.06), while patients with HCV genotype 1b displayed an OR of 2.2 to achieve SVR12 (95% CI 1.0-5.0; *P* = 0.07; see table in Additional file 3). Further analysis of our small cohort revealed that neither the grade of fibrosis, nor the IL28B haplotype, treatment experience, diabetes mellitus type 2, psychiatric disorders, the occurence of a RVR or a reduction of the pegIFN or RBV dose were associated with SVR12 in the univariate analysis (see table in Additional file 3). No independent predictors for SVR12 were identified in the multivariate analysis.

We also examined the frequency and possible consequences of a shortening of the duration of PI medication from the recommended length [15,16]. This analysis revealed that a higher number of patients on BOC treatment (17/37, 45.9%, mean duration of shortening 3.8 weeks, SD = 8.1) compared to patients on TPR treatment (9/65, 13.8%, mean duration of shortening 0.7 weeks, SD = 2.0, *P* < 0.01) reduced the time of protease therapy. The mean shortening of TPR duration was 0.8 weeks (SD = 2.2) in patients with later SVR12 and 0.9 weeks (SD = 2.1) in patients who later experienced a viral breakthrough or relapse (*P* = 0.78, Figure 2B). Patients who experienced virological failure after BOC discontinuation, displayed a mean reduction of BOC medication time of 5.4 weeks (SD = 10.0), compared to 4.0 weeks (SD = 8.1) in BOC patients who achieved SVR12 (*P* = 0.68, Figure 2A). Patients who experienced a relapse discontinued pegIFN and RBV 6.5 weeks prematurely (SD = 13.4) compared to patients who achieved SVR12 (0.4 weeks (SD = 10.7), *P* = 0.03, Figure 2C and D). An early termination of pegIFN/RBV frequently led to treatment failures in patients who qualified for shorter treatment duration (24 weeks for TPR or 28 weeks for BOC, respectively), as well as in patients who were at need for 48 weeks of therapy (Figure 2C and D). Due to the small number of patients in each group, we did not perform a subgroup analysis. The duration of the lead-in phase had no statistically significant impact on the treatment outcome, neither in patients treated with TPR (*P* = 0.30), nor in patients receiving BOC (*P* = 0.68, Figure 2E).

### Side-effects and complications of triple therapy

Detailed information about all side-effects are shown in Table 2. Severe flu-like symptoms were reported by 44 patients (43.1%) and 43 patients (42.2%) showed gastrointestinal symptoms. Patients receiving BOC suffered more often from dysgeusia (24.3%) and fatigue (40.5%) than patients receiving TPR (1.5% and 18.5%, *P* < 0.01 and *P* = 0.02), while TPR based treatment was associated with a high risk for anorectal dyscomfort (36.9% (TPR) versus 2.7% (BOC), *P* < 0.01).

**Table 2:** Side effects and serious adverse events in patients receiving triple therapy

	**Number**	**BOC**	**TPR**	***P-V*** **alue**
	**(N = 102)**	**(N = 37)**	**(N = 65)**	
Grade 3/4 anemia	13 (12.7%)^§^	4 (10.8%)	9 (13.8%)	0.77
Grade 3/4 neutropenia	25 (24.5%)	12 (32.4%)	13 (20%)	0.23
Grade 3/4 thrombopenia	14 (13.7%)	3 (8.1%)	11 (16.9%)	0.25
Flu-like symptoms	44 (43.1%)	20 (54.1%)	24 (36.9%)	0.10
GI disorders	43 (42.2%)	16 (43.2%)	27 (41.5%)	1
Grade 1/2 rash	35 (34.3%)	11 (29.7%)	24 (36.9%)	0.52
Psychiatric disorder	29 (28.4%)^§§^	11 (29.7%)	18 (27.7%)	1
Fatigue	27 (26.5%)	15 (40.5%)	12 (18.5%)	**0.02**
Anorectal dyscomfort	25 (24.5%)	1 (2.7%)	24 (36.9%)^§§§^	**<0.01**
Insomnia	23 (22.5%)	7 (18.9%)	16 (24.6%)	0.63
Bronchopulmonal symptoms	20 (19.6%)	9 (24.3%)	11 (16.9%)	0.44
Dysgeusia	10 (9.8%)	9 (24.3%)	1 (1.5%)	**<0.01**
Immunothyreoiditis	4 (3.9%)	0	4 (6.2%)	0.29
**Serious adverse event**	**Number**	**Comments/Outcome**		
Grade 4 anemia	2	Both patients received blood transfusions		
Grade 4 neutropenia	3	1 patient with neutropenic sepsis; all 3 recovered after dose reduction of peginterferon		
Grade 4 thrombopenia	6	1 patient received a platelet concentrate		
Grade 3 rash	4	TPR was discontinued early in 1 patient		
DRESS/SJS	None	-		
Neurological symptoms	2	Radial nerve paresis (recovery after physiotherapy) and urine incontinency (ongoing)		
Localized infections	3	All fully recovered (balanitis, epididymitis, perspiratory gland abscess)		
Sepsis	4	2 patients recovered; 2 patients died		
Hepatic decompensation	2	Both recovered, but 1 patient died 6 weeks after discontinuation of treatment		
Decompensation of autoimmune disorder	1	First manifestation of autoimmune diabetes mellitus.		
Ileus	2	Fully recovered after surgical intervention (1x) or conservative treatment (1x)		
Cardiac complications	1	Unstable angina pectoris, full recovery after PTCA with stent implantation		

30 SAE occured in 22 individuals. [BOC = boceprevir; TPR = telaprevir; N = number; GI = gastrointestinal; DRESS = drug induced rash and eosinophilia with systemic symptoms; SJS = Stevens Johnson syndrome; PTCA = percutaneous coronary angiography; ^§^ = 5 patients received transfusion; ^§§^ = 9 patients with history of psychiatric illness; ^§§§^ = hemorrhagic proctitis in 5 patients].

Thirty serious adverse events (SAE) occured in 22/102 patients (21.6%). Details on the nature and outcome of the SAE are shown in Table 2.

Tragically, two patients died directly following therapy (mortality 1.9%). Both suffered from cirrhosis (baseline Child-Pugh score: 5 and 7; baseline MELD score: 7 and 12) and the patients received TPR after a four week lead-in phase with pegIFN and RBV. More notable, both patients had known diabetes mellitus type 2 with a baseline HbA1c of 7.1 and 7.8%. The patient with the Child-Pugh score of 7 had also a serum albumin < 35 g/l and a thrombocytopenia < 100,000/µl.

In the univariate analysis advanced age (*P* = 0.02) and existence of liver cirrhosis (*P* < 0.01) were both associated with incidence of a predefined SAE. Furthermore, low platelet count and high INR (*P* < 0.01, each), as well as high ASAT and bilirubin (*P* = 0.04 and 0.01, respectively) were associated with risk for an episode of a SAE.

Age above 50 years (*P* < 0.01), thrombocytes < 100,000/μl (*P* = 0.01), serum albumin < 35 g/l (*P* = 0.04), ASAT > 100 U/l (*P* = 0.03) and bilirubin ≥ 1.2 mg/dl (*P* < 0.01) were significantly associated with the occurence of a SAE (Table 3). Bilirubin ≥ 1.2 mg/dl (OR 13.1; 95%CI 2.1-81.4; *P* < 0.01) and ASAT > 100 U/l (OR 4.6; 95%CI 1.4-15.1; *P* = 0.01) were independent predictors for a SAE in the multivariate analysis.

**Table 3:** Risk factors for occurence of serious adverse events in patients receiving triple therapy

	**No SAE (N = 80)**	**SAE (N = 22)**	***P*-Value**
	**Number (%)**	**Number (%)**	
	**Mean (±SD)**	**Mean (±SD)**	
	**Median (range)**	**Median (range)**	
**Male sex**	50 (62.5%)	13 (59.1%)	0.81
**Age [years]**	46.7 (±12.4)	53.5 (±7.3)	**0.02**
**Age > 50 years**	32 (40%)	16 (72.7%)	**<0.01**
**Liver cirrhosis**	17 (23.9%)	12 (57.1%)	**<0.01**
**Diabetes mellitus type 2**	8 (10%)	5 (22.7%)	0.15
**Psychiatric disorders**	16 (20%)	4 (18.2%)	1
**Exclusion criteria for registration trials**	48 (60%)	17 (77.3%)	0.210
**Drug**			0.45
*Telaprevir*	49 (61.3%)	16 (72.7%)	
*Boceprevir*	31 (38.8%)	6 (27.3%)	
**Baseline laboratory**			
*Hemoglobin [g/dl]*	14.7 (10.3-18.8)	14.6 (12.3-18.0)	0.69
*Leukocytes [x10^9/l]*	6.2 (3.1-13.1)	5.9 (2.7-9.5)	0.79
*Platelets [x10^9/l]*	199.5 (85–377)	127 (48–329)	**<0.01**
*ASAT [U/l]*	49.5 (16–156)	67.5 (26–328)	**0.04**
*ALAT [U/l]*	70 (16–255)	91 (22–271)	0.28
*γGT [U/l]*	62.5 (57–1274)	72 (25–459)	0.18
*Bilirubin [mg/dl]*	0.5 (0.2-1.5)	0.75 (0.2-2.2)	**0.01**
*Albumin [g/l]*	40 (29–50)	39 (25–44)	0.11
*Prothrombin time [INR]*	1.00 (0.9-3.3)	1.13 (1.0-2.3)	**<0.01**
*Creatinine [mg/dl]*	0.8 (0.5-5.8)	0.8 (0.5-1.1)	0.97
*Platelets < 100,000/μl*	5 (6.3%)	6 (27.3%)	**0.01**
*ASAT > 100 U/l*	11 (13.8%)	8 (36.4%)	**0.03**
*Bilirubin ≥ 1.2 mg/dl*	2 (2.5%)	6 (27.3%)	**<0.01**
*Albumin ≤ 35 g/l*	3 (3.8%)	4 (18.2%)	**0.04**
*Prothrombin time [INR] > 1.2*	4 (5.3%)	3 (13.6%)	0.17

[SAE = serious adverse event; N = number; SD = standard deviation; ASAT = aspartate aminotransferase; ALAT = alanine aminotransferase; γGT = gamma-glutamyltransferase; INR = international normalized ratio].

The frequency of SAE in patients with liver cirrhosis was 41.4% (12/29). In the subgroup analysis of patients with liver cirrhosis, median MELD score of patients with a SAE was higher as compared to patients without complications (9 versus 7; *P* < 0.01). Low thrombocytes and elevated INR (*P* < 0.01 and 0.01, respectively) were associated with a SAE, too (see Table in Additional file 4, which displays risk factors for a SAE in cirrhotic patients). The multivariate analysis did not identify any independent predictors for a SAE in the subgroup of cirrhotic patients.

## Discussion

Our study aimed to extend the data obtained by the registration trials of TPR and BOC and to describe our “real-life” experiences of triple therapy in a large cohort of more than 100 patients including “difficult-to-treat” patients (including patients suffering from autoimmune disorders, as well as patients with a major depression) as well as a great number of patients with advanced liver disease. The frequency of F3 or F4 fibrosis (40.2%) was substantially higher as compared to the frequency of bridging fibrosis or cirrhosis in the participants of the registration trials (F3/F4 in 10 to 28% of patients) [3-7]. Of note, almost two-thirds of our patients would have been ineligible for the various registration trials of TPR or BOC [3-7], 15 patients (14.7%) had a history of partial- or null-response in previous treatment and 10 patients were DAA experienced. Astonishingly, these patients displayed a reasonable chance for SVR and the frequency of SVR12 (60.8%) in our “real-life” cohort was only slightly lower as compared to the results from the registration trials [3-7], but still higher than previous “real-life” data on dual treatment with pegIFN/RBV by us and others [17].

At the same time – and in concordance with previous reports [9,10] – we saw a high incidence of SAEs especially in patients with liver cirrhosis and even two fatal outcomes in our cohort. Counterintuitively, patients with psychiatric disorders displayed neither a higher rate of treatment failures, nor a higher risk for the incidence of SAE.

The frequency of treatment failure at week 12 of PI administration was reported to be as high as 29% in previous “real-life” reports [9]. Interestingly, in our cohort only 14.7% of patients experienced a treatment failure until week 12 of PI (13 patients with partial response and 2 patients stopping therapy due to moderate side-effects, Figures 1 and 2). Furthermore, the frequency of SVR was higher as compared to the SVR rate in a most recently published “real-life” cohort [8]. However, our observations might (at least partly) be explained by a higher rate of patients with bridging fibrosis or cirrhosis in these studies, as compared to our cohort [8,9]. On the one hand, the reduction of the pegIFN or RBV dose due to side-effects, as well as an early PI withdrawal (when appropriate) were not associated with lower chances for SVR in our small cohort. On the other hand, an early termination of pegIFN and RBV was determined by us as a risk factor for later relapse. Future prospective studies have to determine whether treatment individualization and de-escalation are indeed a valid option in difficult-to-treat patients to manage side-effects and to achieve a reasonable chance for SVR as seen in this retrospective study.

Our data also indicate, that triple therapy may be a reasonable option for certain DAA-experienced patients, too (Additional file 2). This is an important finding, since the number of DAA-experienced patients will rapidly increase in the future. However, in the future testing of protease inhibitor escape mutations before initiation of re-treatment might be useful in these cases.

Whilst IL28B polymorphism is the strongest pretreatment predictor for SVR in pegIFN/RBV based treatment [18], our results confirm previous reports of limited practical value of IL28B polymorphism for prediction of SVR in patients treated with BOC or TPR [19,20].

Elevated ASAT and bilirubin at baseline were the only independent predictors of SAE in our cohort. However, thrombocytopenia and low serum albumin, which have been identified as the key risk factors for hepatic decompensation and death under triple therapy before [10], were also associated with occurence of a SAE in the univariate analysis in our cohort. Since all deceased patients had cirrhosis and diabetes mellitus type 2, cirrhotic patients with diabetes should be treated with special care since they are most likely to experience severe complications. Furthermore, we recommend that patients who display risk factors for complications, should be referred to an experienced viral hepatitis center.

As a consequence of the treatment complexity of triple therapy with an increased risk for relevant and potentially lethal side-effects, we significantly remodelled procedures at our clinics. Every patient is discussed in a multidisciplinary hepatitis board before HCV triple therapy is initiated. Additionally, every cirrhotic patient is seen by the transplant team before treatment initiation and listed for liver transplantation if deemed necessary.

Our retrospective study has certain limitations. First, in a minority of less than 10% of patients liver cirrhosis was not formally excluded, although none of the patients had any clinical or laboratory signs of liver cirrhosis. Second, we only recorded the fact of RBV dose reduction rather than the individual RBV dose. Since we are associated with a viral hepatitis study center, 81 additional patients (many of them treatment naive) recruited to clinical phase II or III trials during the study period. Finally, our cohort included patients who were previously treated with DAA. Although 50% of the DAA experienced patients achieved SVR12, our study was too small to identify patients who should receive TPR or BOC-based treatment after a virological failure in a DAA based therapy and no assessment of protease excape mutations was performed. However, we believe that this study reflects the “real-life” situation in many large tertiary referral centers and our study provides important learning points in these “challenging-to-treat” patients for other HCV therapy providers worldwide.

## Conclusions

In conclusion, triple therapy with first generation PI provides a reasonable chance for SVR even in “difficult-to-treat” patients, as presented here. However, considering high rates of complications as reported from us and others [9,10], careful patient selection, extensive patient education and precise monitoring are essential, especially in patients with liver cirrhosis.

## Competing interests

Malte H. Wehmeyer has served as a speaker for BMS. Sabine Jordan, Olaf Degen, Martina Sterneck, Jan van Lunzen, Ansgar W. Lohse, Julian Schulze zur Wiesch and Stefan Lüth have served as speakers for Roche, Janssen-Cilag and MSD. Annette Hennigs has served as a speaker for Janssen-Cilag. Friederike Eißing, Claudia Röder, Anja Hüfner, Sandra Hertling and Stefan Schmiedel declare no conflict of interest.

## Authors’ contributions

MHW drafted the originial manuscript, contributed to study design, performed the statistical analysis, interpreted the results and collected the data; FE performed additional statistical analysis and collected the data. SJ, AHe, OD, AHü, SH, SS and MS collected the data; CR contributed to the study design; JvL contributed to study design and data collection; AWL critically revised the manuscript; JSzW and SL contributed to study design, collected data and critically revised the manuscript. All authors read and approved the final manuscript.

## Pre-publication history

The pre-publication history for this paper can be accessed here:

http://www.biomedcentral.com/1471-230X/14/87/prepub

## Supplementary Material

Additional file 1 Virological and adverse events definitions, grading of laboratory events and further information on the DAA experienced patients. Click here for file

Additional file 2 Course of therapy in DAA-experienced patients, as well as in patients with special comorbidities. Click here for file

Additional file 3 Evaluation of predictors for SVR12. Click here for file

Additional file 4 Evaluation of risk factors for SAE in cirrhotic patients. Click here for file
