# Supplementary material for: Safety and efficacy of protease inhibitor based combination therapy in a single-center “real-life” cohort of 110 patients with chronic hepatitis C genotype 1 infection
Source: BMC Gastroenterol. 2014 May 5;14:87. doi: 10.1186/1471-230X-14-87 (PMC4102246; doi:10.1186/1471-230X-14-87)
Supplement: Additional file 1 — Virological and adverse events definitions, grading of laboratory events and further information on the DAA experienced patients. [file 1471-230X-14-87-S1.doc]

**Virological and adverse event definitions, grading of laboratory events**

SVR was defined as undetectable viral load 12 weeks after last RBV dose (SVR12). A relapse was therefore defined as any detectable HCV viral load during the first 12 weeks after completion of treatment. Breakthrough was assumed, if a former undetecable viral load became detectable again during therapy and partial response was defined as discontinuation of triple therapy after applying futility rules for PI [14,15]. Rapid virological response (RVR) was defined as undetectable viral load 4 weeks after first PI dosage.

Grade 3 anemia was defined as hemoglobin concentration (Hb) from 7.9 to 6.5 g/dl and grade 4 anemia was defined as Hb below 6.5 g/dl. Grade 3 and 4 neutropenia was defined as a neutrophilic granolocytes count from 750 to 500/µl (G3) or below 500 neutrophils/µl (G4) respectively. Grade 3 and 4 thrombopenia were defined as a platelet count between 50,000 and 25,000/µl (G3) or below 25,000/µ (G4).

Serious adverse events (SAE) were defined as any condition leading to prolonged invalidity, hospitalization or death, as well as any grade 4 laboratory events and severe skin reactions (grade 3 rash, DRESS (drug induced rash with eosinophilia and systemic symptoms), Stevens-Johnson syndrome).

**Additional information on the DAA experienced patients**

In total, 10/55 patients who were HCV treatment experienced, have received direct acting antivirals (DAA) in the course of a previous therapy. All were participants in clincial trials and no patient received TPR or BOC in the course of the clinical trial. Most of the DAA experienced patients were man (8/10) and 4/10 suffered from liver cirrhosis. One half of the patients (5/10) experienced a virological failure during or following the DAA based treatement more than six months prior to the start of the current triple therapy. However, the remaining 5 patients (patient number 2, 3, 6, 7 and 8 in the upper figure in **Additional file 2**) started the triple therapy with TPR directly following the virological failure of the DAA based treatment regimen or the discontinuation of the trial (patient 2), respectively.
