# Supplementary material for: Safety and efficacy of protease inhibitor based combination therapy in a single-center “real-life” cohort of 110 patients with chronic hepatitis C genotype 1 infection
Source: BMC Gastroenterol. 2014 May 5;14:87. doi: 10.1186/1471-230X-14-87 (PMC4102246; doi:10.1186/1471-230X-14-87)
Supplement: Additional file 2 — Course of therapy in DAA-experienced patients, as well as in patients with special comorbidities. [file 1471-230X-14-87-S2.pdf]

A DAA experienced patients

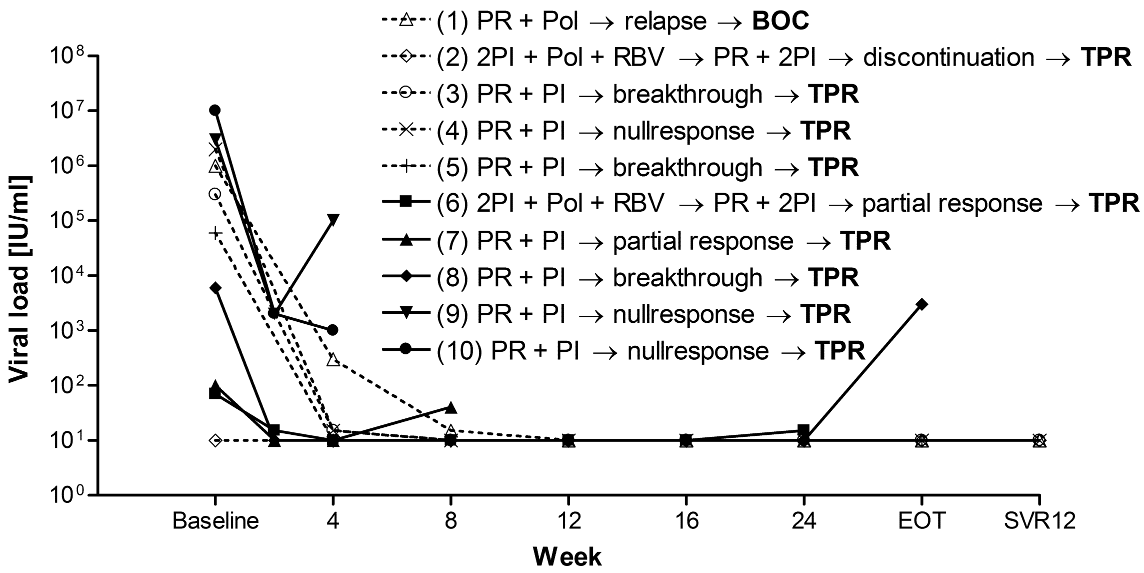

B Selected patients with special comorbidities

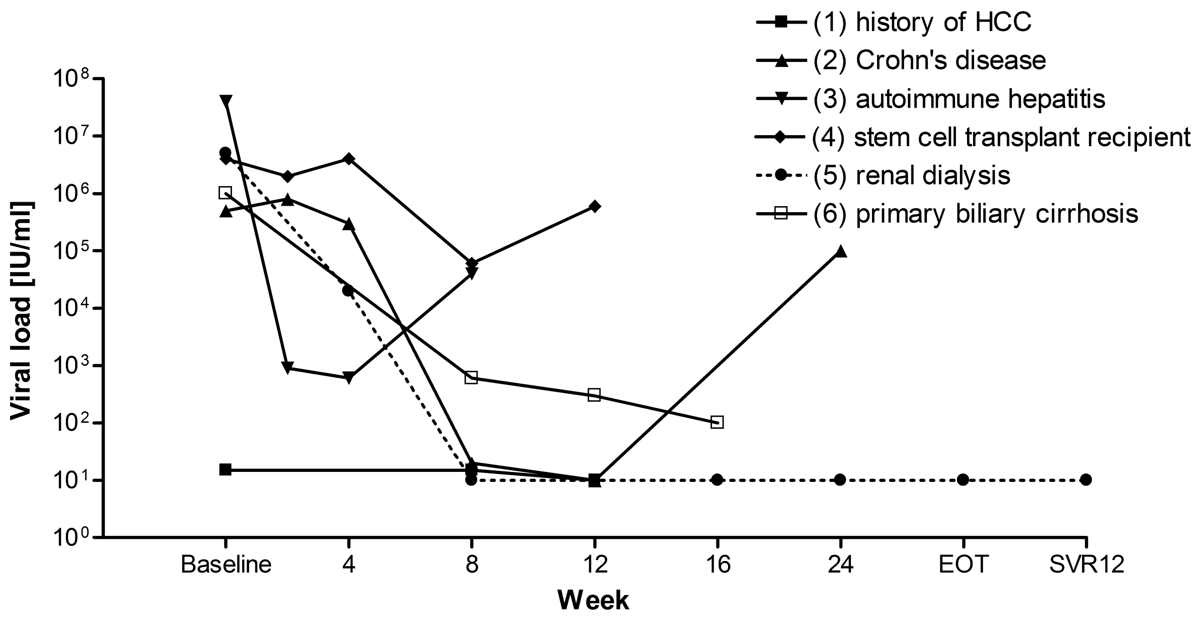

Additional figure 1. Individual courses of DAA experienced patients [A] and patients with special comorbidities [B]. Each line represents the viral load of one individual patient. Patients who have achieved SVR12 are represented by dotted lines. Figure legends identify medication and outcome of prior therapy, as well as the protease inhibitor

used in triple therapy [A], or a concomitant disease, respectively [B]. [DAA = direct acting antiviral; BOC = boceprevir; TPR = telaprevir; EOT = end of treatment; SVR12 = sustained virological response 12 weeks after last ribavirin dose; PR = pegylated interferon in combination with ribavirin; 2PI = patient received a combination of two different protease inhibitors; Pol = polymerase inhibitor; RBV = ribavirin; PI = protease inhibitor]
