# Supplementary material for: Safety and efficacy of protease inhibitor based combination therapy in a single-center “real-life” cohort of 110 patients with chronic hepatitis C genotype 1 infection
Source: BMC Gastroenterol. 2014 May 5;14:87. doi: 10.1186/1471-230X-14-87 (PMC4102246; doi:10.1186/1471-230X-14-87)
Supplement: Additional file 3 — Evaluation of predictors for SVR12. [file 1471-230X-14-87-S3.doc]

|  | **SVR12**  (N = 62) | **Treatment failure**  (N = 40) | **OR**  (95%CI) | ***P*-Value** |
| --- | --- | --- | --- | --- |
| **Male sex** | 38 (61.3%) | 25 (62.5%) | 1.0 (0.4-2.2) | 1 |
| **Drug** |  |  |  |  |
| *Telaprevir* | 40 (64.5%) | 25 (62.5%) | 1.1 (0.5-2.5) | 1 |
| *Boceprevir* | 22 (35.5%) | 15 (37.5%) | 0.9 (0.4-2.1) | 1 |
| **Exclusion criteria for appropriate phase III trial** | 37 (59.7%) | 28 (70%) | 0.6 (0.3-1.5) | 0.30 |
| **Diabetes mellitus type 2** | 6 (9.7%) | 7 (17.5%) | 0.5 (0.2-1.6) | 0.36 |
| **Psychiatric disorder** | 11 (17.7%) | 9 (22.5%) | 0.7 (0.3-2.0) | 0.61 |
| **Treatment naive** | 27 (43.5%) | 20 (50%) | 0.8 (0.3-1.7) | 0.55 |
| **Prior treatment** |  |  |  |  |
| *Relapse* | 18 (29.0%) | 7 (17.5%) | 1.9 (0.7-5.2) | 0.24 |
| *Null/partial response* | 7 (11.3%) | 8 (20%) | 0.5 (0.2-1.5) | 0.26 |
| *Breakthrough* | 4 (6.5%) | 2 (5%) | 1.3 (0.2-7.5) | 1 |
| *Discontinuation^§^* | 3 (4.8%) | 2 (5%) | 1.0 (0.2-6.1) | 1 |
| *Unknown outcome* | 3 (4.8%) | 1 (2.5%) | 2.0 (0.2-19.8) | 0.65 |
| **DAA experienced** | 5 (8.1%) | 5 (12.5%) | 0.6 (0.2-2.3) | 0.51 |
| **RVR** | 27 (43.5%) | 11 (27.5%) | 2.0 (0.9-4.8) | 0.14 |
| **Genotype** |  |  |  |  |
| *Genotype 1a* | 19 (30.6%) | 20 (50%) | 0.4 (0.2-1.0) | 0.06 |
| *Genotype 1b* | 37 (59.7%) | 16 (40%) | 2.2 (1.0-5.0) | 0.07 |
| *No subtype provided* | 5 (8.1%) | 4 (10%) | 0.8 (0.2-3.1) | 1 |
| *Unknown* | 1 (1.6%) | 0 | - | 1 |
| **IL28B** (N = 70) |  |  |  |  |
| *C/C* | 10 (16.1%) | 6 (15%) | 1.4 (0.5-4.3) | 0.58 |
| *C/T* | 22 (35.5%) | 20 (50%) | 0.7 (0.3-1.9) | 0.62 |
| *T/T* | 7 (11.3%) | 5 (12.5%) | 1.1 (0.3-4.0) | 1 |
| **Stage of fibrosis** (N = 92) |  |  |  |  |
| *No to mild fibrosis (F0-F2)* | 34 (54.8%) | 17 (42.5%) | 1.9 (0.8-4.4) | 0.14 |
| *Bridging fibrosis (F3)* | 6 (9.7%) | 6 (15%) | 0.5 (0.1-1.8) | 0.33 |
| *Liver cirrhosis* | 15 (24.2%) | 14 (35%) | 0.6 (0.3-1.5) | 0.36 |
| **Dose reduction pegIFN** | 24 (38.7%) | 11 (27.5%) | 1.7 (0.7-3.9) | 0.29 |
| **Dose reduction RBV** | 35 (56.5%) | 17 (42.5%) | 1.8 (0.8-3.9) | 0.22 |
| **Anemia ≥ grade 2** | 29 (46.8%) | 14 (35%) | 1.6 (0.7-3.7) | 0.31 |

**Additional table 1. Evaluation of possible predictor variables for sustained virological response in triple therapy.** [N = Number; SVR12 = sustained virological response 12 weeks after last ribavirin dose; OR = odds ratio; 95%CI = 95%-confidence interval; DAA = direct acting antivirals; RVR = rapid virological response; IL28B = interleukin-28B polymorphism; PI = protease inhibitor; pegIFN = pegylated interferon alfa; RBV = ribavirin; ^§^ = discontinuation of prior therapy due to side-effects]
