# Supplementary material for: Safety and efficacy of protease inhibitor based combination therapy in a single-center “real-life” cohort of 110 patients with chronic hepatitis C genotype 1 infection
Source: BMC Gastroenterol. 2014 May 5;14:87. doi: 10.1186/1471-230X-14-87 (PMC4102246; doi:10.1186/1471-230X-14-87)
Supplement: Additional file 4 — Evaluation of risk factors for SAE in cirrhotic patients. [file 1471-230X-14-87-S4.doc]

|  | **No SAE** (N = 17)  Number (%)  Mean (±SD) | **SAE** (N = 12)  Number (%)  Mean (±SD) | ***P*-Value** |
| --- | --- | --- | --- |
| **Male sex** | 12 (70.6%) | 8 (66.7%) | 1 |
| **Age [years]** | 48.7 (±11.7) | 53.3 (±7.8) | 0.24 |
| **Age > 50 years** | 8 (47.1%) | 9 (75%) | 0.25 |
| **Diabetes mellitus type 2** | 3 (17.6%) | 5 (41.7%) | 0.22 |
| **Psychiatric disorders** | 3 (17.6%) | 2 (16.7%) | 1 |
| **Exclusion criteria for registration trials** | 14 (82.4%) | 9 (75%) | 0.67 |
| **Drug** |  |  | 1 |
| *Telaprevir* | 13 (76.5%) | 9 (75%) |  |
| *Boceprevir* | 4 (23.5%) | 3 (25%) |  |
| *Child-Pugh score > 5* | 2 (11.8%) | 5 (41.7%) | 0.09 |
| *Child-Pugh score > 6* | 0 | 2 (16.7%) | 0.16 |
| *MELD score* | 7 (6-9) | 9 (6-12) | **<0.01** |
| *MELD score > 10* | 0 | 4 (33.3%) | **0.02** |
| **Baseline laboratory** |  |  |  |
| *Hemoglobin [g/dl]* | 14.4 (11.2-17.3) | 13.95 (12.3-18.0) | 0.84 |
| *Leukocytes [x10^9/l]* | 5.9 (3.1-12.4) | 5.35 (2.7-9.5) | 0.86 |
| *Platelets [x10^9/l]* | 133 (85-239) | 86 (48-203) | **<0.01** |
| *ASAT [U/l]* | 55 (19-138) | 68.5 (30-138) | 0.39 |
| *ALAT [U/l]* | 64 (26-197) | 82.5 (22-213) | 0.71 |
| *γGT [U/l]* | 96 (30-523) | 102.5 (32-459) | 0.71 |
| *Bilirubin [mg/dl]* | 0.6 (0.3-1.5) | 0.9 (0.4-2.2) | 0.11 |
| *Albumin [g/l]* | 39.2 (±4.7) | 37.5 (25-44) | 0.26 |
| *Prothrombin time [INR]* | 1.02 (0.96-1.27) | 1.145 (0.96-1.46) | **0.01** |
| *Creatinine [mg/dl]* | 0.8 (0.6-1.2) | 0.8 (0.5-1.1) | 0.75 |
| *Platelets < 100,000/µl* | 3 (17.6%) | 6 (50%) | 0.10 |
| *ASAT > 100 U/l* | 3 (17.6%) | 4 (33.3%) | 0.40 |
| *Bilirubin ≥ 1.2 mg/dl* | 1 (5.8%) | 5 (41.7%) | 0.06 |
| *Albumin ≤ 35 g/l* | 2 (11.8%) | 3 (25%) | 0.62 |
| *Prothrombin time [INR] > 1.2* | 2 (11.8%) | 2 (16.7%) | 1 |

**Additional table 2. Risk factors for occurence of serious adverse events during triple therapy in patients with liver cirrhosis.** [SAE = serious adverse event; N = number; SD = standard deviation; MELD = model for end stage liver disease; ASAT = aspartate aminotransferase; ALAT = alanine aminotransferase; γGT = gamma-glutamyltransferase; INR = international normalized ratio]
